# Mechanical Performance and Strength Prediction of Non-Steam-Curing UHPC Considering Coupled Temperature-Constituent Effects

**DOI:** 10.3390/ma19214521

**Published:** 2026-10-27

**Authors:** Changjin Tian, Yefeng Du, Ran An, Youzhi Wang, Xiaoning Zhang, Lin Teng

**Affiliations:** 1 School of Civil Engineering, Qilu Institute of Technology, Jinan 250200, China; 17862990729@163.com (C.T.); t-lin1001@qlit.edu.cn (L.T.); 2 China Construction Infrastructure Corp. Ltd., Beijing 100029, China; 3 Department of Civil and Environmental Engineering, The Hong Kong Polytechnic University, Hong Kong, China; yefeng.du@polyu.edu.hk; 4 School of Civil Engineering, Shandong University, Jinan 250061, China; wangyouzhi@sdu.edu.cn (Y.W.); sdjtuzxn@163.com (X.Z.)

**Keywords:** non-steam-curing UHPC, curing temperature, mineral admixture content, mechanical properties, predictive model

## Abstract

Non-steam-curing ultra-high-performance concrete (UHPC) is a promising material for in situ bridge deck rehabilitation; however, its strength development under variable curing temperatures remains insufficiently understood. This study investigated the coupled effects of curing temperature and constituent dosage on the mechanical performance of non-steam-curing UHPC. Compressive and flexural strength tests were conducted on mixtures incorporating different dosages of silica fume, microspheres, and steel fibers. Based on the experimental results, two predictive models were developed to describe the time-dependent strength evolution of UHPC at the optimal mixture proportion and the later-age strength as a function of curing temperature and constituent dosage. The results showed that silica fume had a significant influence on early-age strength, but its effect was strongly dependent on curing temperature and age. At low curing temperatures, silica fume initially reduced early-age strength, whereas its contribution became positive with prolonged curing. When the microsphere dosage exceeded 55 kg/m^3^, early-age strength decreased with increasing microsphere content; however, this adverse effect gradually weakened and eventually became beneficial as curing temperature and curing age increased. Increasing the steel fiber dosage from 40 to 160 kg/m^3^ consistently improved both compressive and flexural strengths, whereas further increases produced only marginal additional enhancement. The proposed models captured the temperature-dependent strength development of non-steam-curing UHPC and provide a quantitative basis for mixture optimization and construction-stage strength prediction in bridge rehabilitation applications.

## 1. Introduction

The integration of ultra-high-performance concrete (UHPC) with orthotropic steel decks (OSDs) has been widely recognized as an effective strategy for mitigating fatigue cracking and pavement layer deterioration in conventional steel bridge decks, showing considerable potential for bridge strengthening and rehabilitation applications [1,2,3,4,5]. This approach is particularly attractive for the rehabilitation of in-service bridges, where the overlay material is required to develop sufficient early-age strength while maintaining long-term durability. Conventional UHPC typically relies on steam or autoclave curing to accelerate early hydration and enhance early-age mechanical performance. However, these curing regimes inevitably increase production cost and energy consumption [6,7,8]. Moreover, steam or autoclave curing is unsuitable for retrofitting existing steel bridges because large thermal gradients and repeated heating–cooling cycles may adversely affect the stress state and service performance of existing OSD systems [9,10]. Consequently, non-steam-curing UHPC emerges as a highly promising deck overlay material for the strengthening and rehabilitation of in-service bridges.

Extensive research worldwide has focused on mix design, internal curing strategies, and microstructural optimization of non-steam-curing UHPC [7,11]. In terms of binder optimization, highly reactive mineral admixtures, including silica fume, metakaolin, and ground granulated blast-furnace slag, have been incorporated to improve particle packing, promote pozzolanic reactions, and densify the cementitious matrix [12,13,14,15,16,17,18]. Nanoscale materials and optimized water-to-binder ratios have also been used to accelerate early hydration and refine the pore structure of UHPC [19,20,21,22,23]. In addition, high-range water-reducing agents and chemical activators can further promote the formation of calcium-silicate-hydrate C–S–H gels, thereby improving early-age strength development under non-steam-curing conditions [24,25].

For internal curing, lightweight expansive materials, pre-saturated lightweight aggregates, or superabsorbent polymers have been used to provide gradual moisture release, mitigating autogenous shrinkage and improving the hydration environment, thus ensuring sufficient reaction of the cementitious matrix under non-steam-curing conditions [26,27,28,29,30,31]. These studies also indicate that the large amount of cementitious materials in UHPC generates substantial hydration heat, which raises the internal temperature of the UHPC matrix and in turn accelerates the hydration rate, potentially enhancing early-age strength development [8,32,33,34,35]. However, compared with standard or high-temperature steam curing, the influence of this temperature rise on the early mechanical performance of UHPC remains insufficiently understood [36]. In northern China, where winter arrives early and ambient temperature and humidity are low, UHPC overlays for in situ rehabilitation of existing steel bridges are inevitably cured at relatively low temperatures [37]. To ensure adequate early-age strength, facilitate faster formwork turnover, and shorten construction schedules, contractors often adopt mild thermal insulation or low-level heating measures. Yet, excessive temperature elevation may adversely affect long-service-life steel bridge decks. Consequently, moderate curing temperatures may provide an optimal balance for non-steam-curing UHPC overlays in bridge rehabilitation projects [23,38,39]. A systematic understanding of the temperature-dependent mechanical development of UHPC, especially the interaction between curing temperature and key functional constituents, is essential for mixture optimization and construction-stage performance control.

However, existing studies have mainly focused on mixture optimization, internal curing, or curing temperature as separate factors. The coupled effects of curing temperature and constituent dosage on the mechanical behavior of non-steam-curing UHPC remain insufficiently clarified. In particular, the temperature-dependent roles of silica fume, microspheres, and steel fibers in early-age and later-age strength development have not been systematically quantified. Moreover, predictive models that can describe strength evolution under different curing temperatures and constituent dosages are still limited.

In this study, the temperature sensitivity of non-steam-curing UHPC was investigated through compressive and flexural strength tests on mixtures prepared with different curing temperatures and dosages of silica fume, microspheres, and steel fibers. The effects of these key constituents on strength development were analyzed in relation to curing temperature and curing age. Based on the experimental results, two predictive models were developed: one for describing the time-dependent evolution of UHPC mechanical properties considering temperature effects, and the other for predicting later-age strength as a function of curing temperature and constituent dosage. The findings provide a quantitative basis for optimizing non-steam-curing UHPC mixtures and predicting construction-stage strength development in bridge rehabilitation applications.

## 2. Compressive and Flexural Strength Tests

### 2.1. Raw Materials of UHPC

**Figure 1:**
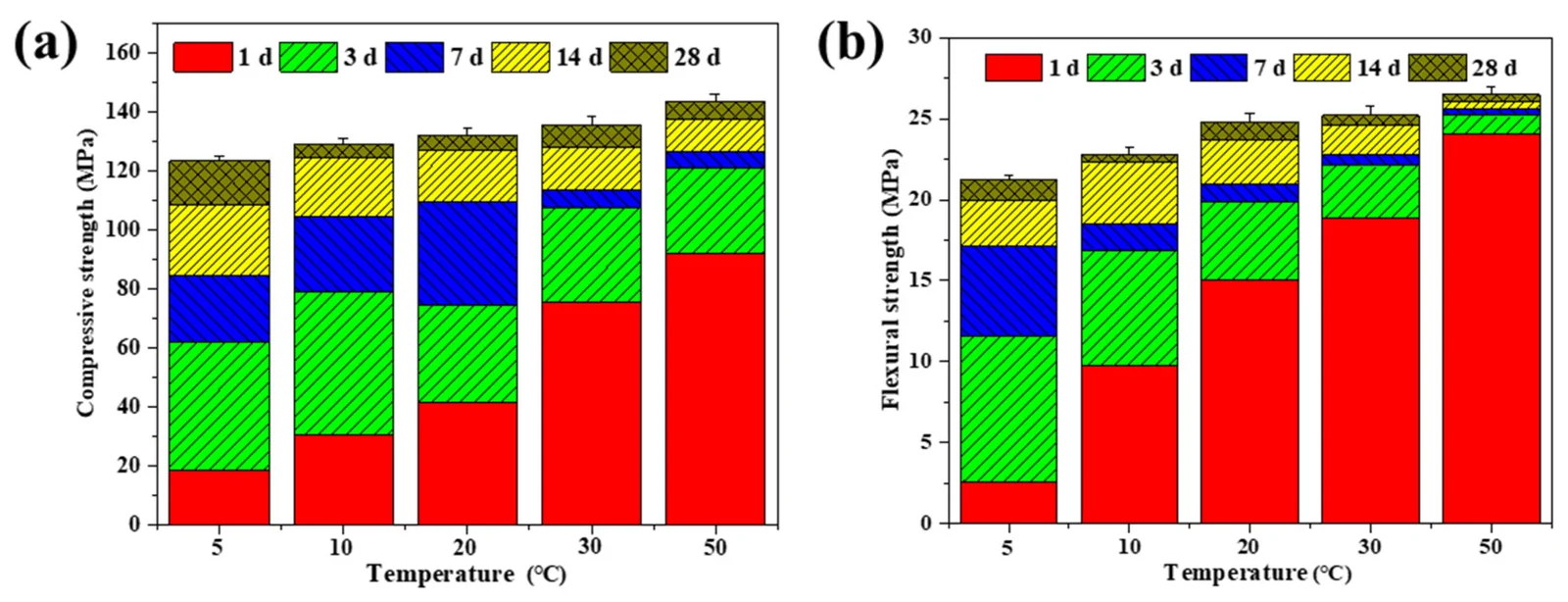
Influence of curing temperature on the mechanical properties of UHPC: (**a**) compressive strength; (**b**) flexural strength.

The non-steam-curing UHPC was produced using cement, fly ash microspheres (hereafter referred to as microspheres), silica fume, quartz sand, a polycarboxylate-based high-range water-reducing admixture, steel fibers, an expansive agent, and internal curing materials, as shown in Figure 1. The expansive agent was primarily magnesium oxide (MgO), while the internal curing material was a superabsorbent polymer (SAP). The expansive agent consisted of micron-sized and nano-sized magnesium oxide (MgO) powders, both with a purity of 99.9%. Their average particle sizes were 4.0 and 0.4 μm, and their specific surface areas were 13 and 19 m^2^/g, respectively. The internal curing material was a superabsorbent polymer (SAP-40), with a particle size of 150–450 μm and a water absorption capacity of 55 g/g. Additional specifications of the other raw materials are provided in reference [40].

### 2.2. Specimen Preparation

Table 1 presents the optimal mix proportion of UHPC, with the detailed determination method available in reference [40]. The preparation procedure was as follows: all powder materials (cement, microspheres, silica fume, expansive agent, and internal curing material) and quartz sand were dry-mixed in a mixer for 3 min. Then, water and the water-reducing admixture solution were added and mixed for 5 min. Steel fibers were gradually introduced into the mixer while stirring, followed by an additional 6 min of mixing. After measuring workability, the fresh UHPC was cast into molds, leveled with a trowel, and covered with plastic film. After casting, the specimens were covered with plastic film and immediately placed in temperature-controlled curing environments at 5, 10, 20, 30, or 50 °C. The specimens were kept in their molds for the first 24 h. Specimens designated for the 1-day strength tests were tested at 24 h, while the remaining specimens were demolded and returned to their respective curing environments until the specified testing ages.

The MgO expansive agent consisted of two particle-size fractions, each incorporated at 2% of the original cementitious material content. The MgO powders partially replaced cement, silica fume, and microspheres, whereas SAP was externally added at 0.2%. The effective water-to-binder ratio was 0.18, with an additional water-to-binder ratio of 0.05 for internal curing, resulting in a total water-to-binder ratio of 0.23. The mixture proportions were calculated based on the original cementitious material content.

### 2.3. Test Methods and Experimental Program

Compressive and flexural strength tests of non-steam-curing UHPC were conducted following the Standard for Test Methods of Concrete Physical and Mechanical Properties (GB/T 50081-2019) [41]. Compressive strength specimens were cubes with dimensions of 100 × 100 × 100 mm, while flexural strength specimens were prisms measuring 100 × 100 × 400 mm. Specimens were cured to the designated ages before testing. The tests were performed using a TSY-2000 electro-hydraulic pressure testing machine (Zhejiang Luda Machinery Instrument Co., Ltd., Shaoxing, China) for compressive strength and a WAW-100B servo-controlled universal testing machine (Jinan Liling Testing Machine Co., Ltd., Jinan, China) for flexural strength. The loading rate for the compressive test was 1.5 MPa/s, and for the flexural test, it was 0.05 mm/min; both tests continued until specimen failure. For each experimental condition, three specimens were tested, and the mean value was reported. The standard deviations of the 28-day compressive and flexural strengths are additionally provided in Figure 1 to characterize the variability of the experimental results. To investigate the effects of curing temperature and mineral admixture content on UHPC mechanical properties, curing temperatures of 5, 10, 20, 30, and 50 °C were selected. Silica fume dosages were 40, 80, 120, 160, and 180 kg/m^3^; microsphere dosages were 30, 55, 110, 165, and 220 kg/m^3^; and steel fiber dosages were 40, 80, 120, 160, 200, and 220 kg/m^3^. For the silica fume and microsphere series, the respective dosages were varied by replacing an equivalent mass of cement, thereby maintaining a constant total binder content within each series. Compressive and flexural strengths were measured at curing ages of 1, 3, 7, 14, and 28 days.

## 3. Experimental Results and Discussion

### 3.1. Effect of Curing Temperature on the Early-Age Mechanical Properties of Non-Steam-Curing UHPC

**Figure 2:**
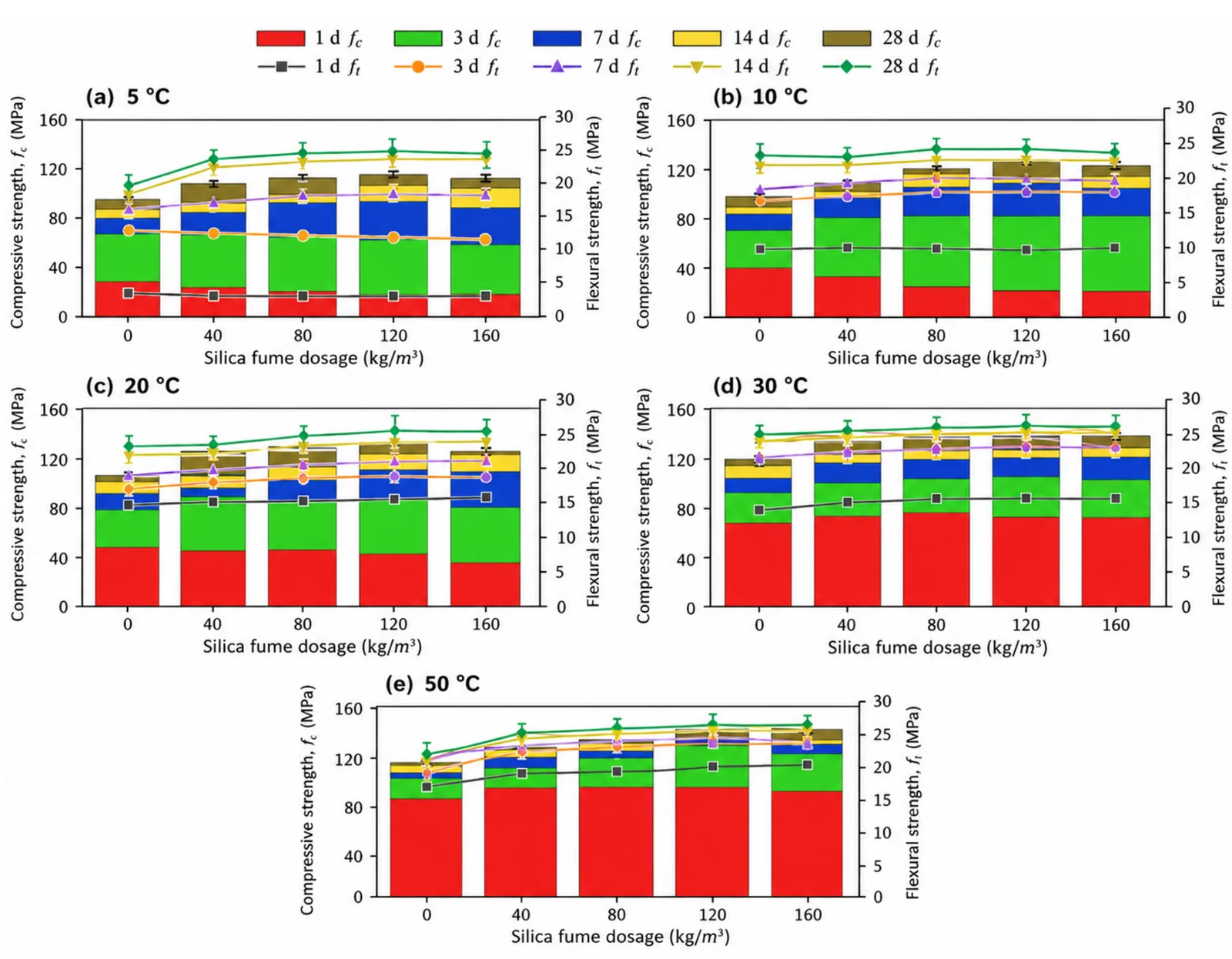
Effect of silica-fume dosage on the mechanical properties of non-steam-curing UHPC: (**a**) 5 °C; (**b**) 10 °C; (**c**) 20 °C; (**d**) 30 °C; (**e**) 50 °C.

Figure 2 illustrates the compressive failure patterns of non-steam-curing UHPC specimens after 7 days of curing at different temperatures. As shown, all specimens exhibited pronounced lateral deformation at the center of the top surface and an evident increase in side cracking. The specimens remained largely intact after failure, without spalling, and the interval from crack initiation to ultimate failure was relatively long. This behavior is attributed to the interfacial bonding and frictional resistance provided by the steel fibers, which impart excellent compressive toughness and enhance the plastic deformation capacity of the UHPC matrix. Moreover, as curing temperature increased, surface spalling progressively diminished and the deformation of the loaded face decreased, indicating that higher curing temperatures improved the surface integrity and resistance to localized deformation under compression.

Figure 1 presents the compressive and flexural strengths of non-steam-curing UHPC at different curing temperatures and ages. Overall, both strengths increased markedly with rising curing temperature, but the strength gap between different temperatures gradually narrowed as the curing age increased. As shown in Figure 1a, the 1-day compressive strength at 50 °C was approximately 400% higher than that at 5 °C, whereas at 28 days the difference decreased to only 14.2%. Moreover, the rate of strength gain with curing age declined significantly as the curing temperature increased. At 5 °C, the 14-day compressive strength was 511.1% higher than the 1-day value, while at 50 °C it increased by only 36.7% over the same period. Flexural strength exhibited the same trend. Notably, the growth of flexural strength beyond early ages slowed more sharply with increasing temperature: at 50 °C, the 28-, 14-, 7-, and 3-day flexural strengths exceeded the 1-day value by only 9.5%, 4.2%, 3.8%, and 3.4%, respectively. The different rates of strength development under various curing temperatures may be associated with temperature-dependent hydration behavior. Previous studies have indicated that elevated curing temperatures can accelerate cement hydration and promote the pozzolanic reactions of mineral admixtures, contributing to higher early-age strength. However, accelerated early hydration may also affect subsequent hydration processes, which could partly explain the smaller later-age strength gain observed at 50 °C than at 30 °C. By contrast, hydration generally proceeds more slowly at lower curing temperatures, allowing strength to develop more gradually with curing age. Consequently, the experimentally observed strength differences between curing temperatures narrowed progressively as curing age increased.

### 3.2. Effect of Silica Fume on the Early-Age Mechanical Properties of Non-Steam-Curing UHPC

Figure 2 illustrates the influence of silica fume on the compressive and flexural strengths of UHPC. According to Table 1 and Figure 2, silica fume can markedly enhance the 28-day mechanical properties of UHPC under various curing temperatures, and this strengthening effect becomes more pronounced as the curing temperature rises—especially at early ages. At 5 °C, increasing the silica-fume dosage reduced the early-age compressive and flexural strengths, whereas the 28-day strengths increased as the dosage increased up to 120 kg/m^3^. For example, under a curing temperature of 5 °C, when the silica-fume dosage increases from 40 kg/m^3^ to 80 kg/m^3^, the 3-day compressive strength decreases from 22.4 MPa to 18.2 MPa, whereas the 28-day compressive strength slightly increases from 108.5 MPa to 110.5 MPa. Similarly, the 3-day flexural strength decreases from 2.8 MPa to 2.7 MPa, while the 28-day flexural strength increases from 22.5 MPa to 23.1 MPa. At the same silica-fume dosage, the 28-day compressive and flexural strengths of the S120T50 specimens are 26.8% and 10.0% higher, respectively, than those of the S120T5 specimens. These results indicate that elevated curing temperature is beneficial for activating the pozzolanic activity of silica fume and for promoting the reaction between silica fume and Ca(OH)_2_. Compared with flexural strength, the increase in curing temperature has a more significant positive impact on compressive strength.

Furthermore, when the silica-fume dosage is excessively high (>120 kg/m^3^), the mechanical properties exhibit a slight decrease even under elevated curing temperatures. For instance, at a curing age of 7 days, the S160T50 specimens show compressive and flexural strengths of 140.2 MPa and 23.9 MPa, respectively, which are 1.8% and 2.0% lower than those of S120T50 at the same age. The beneficial influence of silica fume at appropriate dosages may be attributed to its particle-filling effect and pozzolanic reactions, as suggested by previous studies. With increasing temperature, the pozzolanic activity of silica fume is more easily activated. Due to the high reactivity of the amorphous silica within the fume, pozzolanic reactions occur mainly at early ages, so the temperature effect on silica fume is more significant during the early stage, leading to a marked improvement in the early-age mechanical properties of UHPC. On the other hand, because of its very large specific surface area, excessive silica fume absorbs a substantial amount of water from the UHPC matrix, which hinders the hydration of the cementitious materials and reduces the formation of C-S-H gel. Even though high curing temperatures can stimulate the pozzolanic reactivity of silica fume, the reduction in available water still weakens both the pozzolanic reaction and cement hydration. Therefore, when the silica-fume dosage is too high, the mechanical properties of the system are adversely affected. Under low-temperature curing conditions, the silica-fume dosage should be carefully controlled to avoid reductions in early-age mechanical performance.

### 3.3. Effect of Microspheres on the Early-Age Mechanical Properties of Non-Steam-Curing UHPC

**Figure 3:**
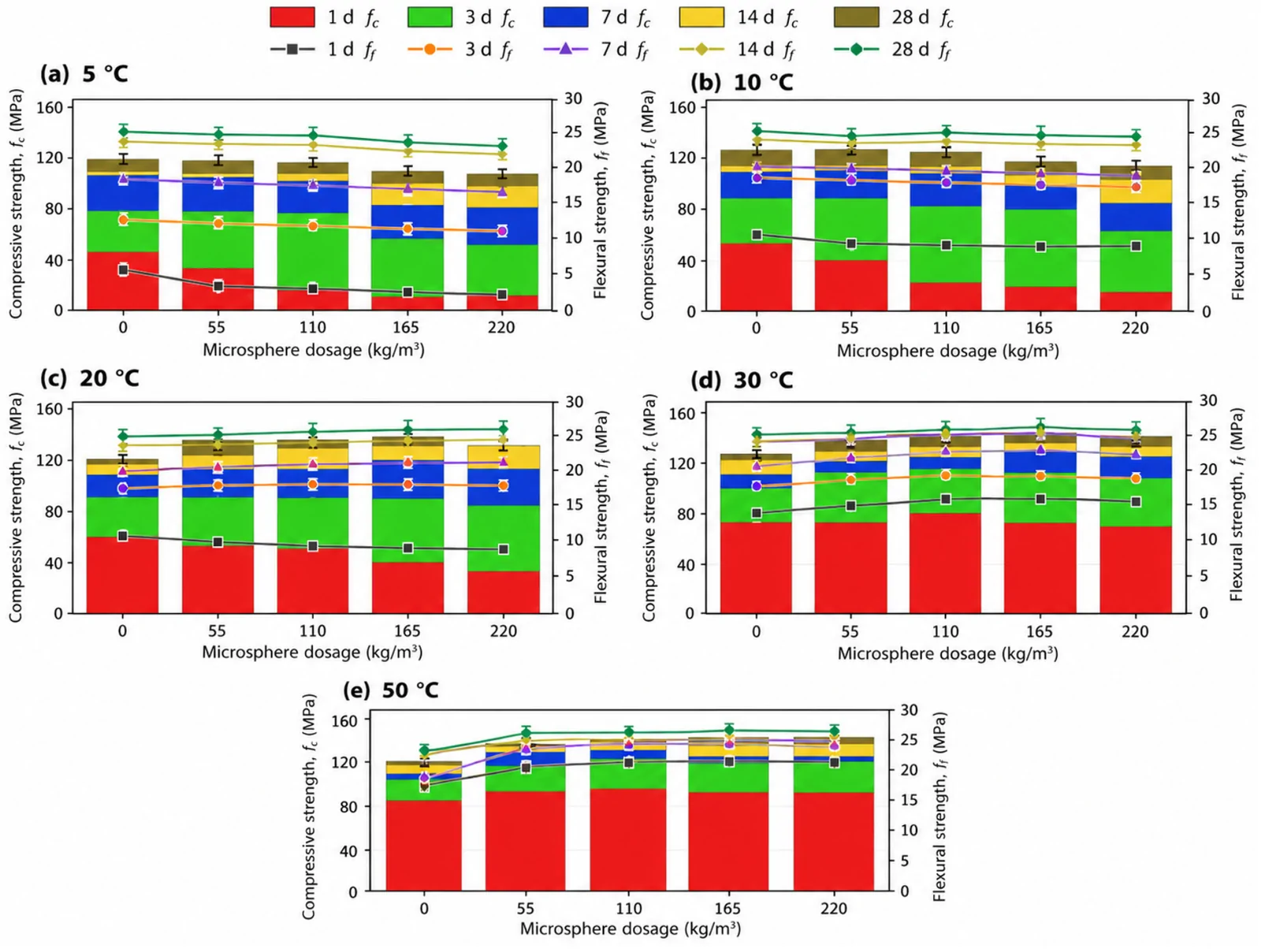
Effect of microsphere dosage on the mechanical properties of non-steam-curing UHPC: (**a**) 5 °C; (**b**) 10 °C; (**c**) 20 °C; (**d**) 30 °C; (**e**) 50 °C.

Figure 3 illustrates the effect of microsphere dosage on the mechanical properties of UHPC at different curing temperatures. According to Table 1 and Figure 3, under low-temperature curing conditions, increasing the microsphere dosage generally reduces both compressive and flexural strengths, particularly at early curing ages (1 and 3 days). The reduction becomes less pronounced at later hydration stages. For example, at 1-day curing, C55T5 exhibits compressive and flexural strengths of 32.7 MPa and 3.1 MPa, respectively, whereas C165T5 shows 15.4 MPa and 2.4 MPa, representing decreases of 52.9% and 22.6%. As curing temperature and age increase, the reduction in UHPC strength with increasing microsphere content gradually disappears, and the “negative effect” of microspheres transitions to a “positive effect.” This phenomenon arises because replacing part of the cement with microspheres reduces the relative amount of cement in the UHPC matrix, slowing the hydration rate of the cement-microsphere system. Consequently, the early-age hydration products cannot form a dense network, lowering early-age strength. Under low-temperature conditions, this effect is more pronounced because the secondary pozzolanic reaction of microspheres primarily occurs at later stages. Over time, even at low temperatures, some microspheres gradually react with Ca(OH)_2_ from cement hydration, forming additional C-S-H gel that fills the voids and refines pore size, thereby improving late-age mechanical properties. The more favorable strength response at higher curing temperatures may be associated with the temperature-dependent pozzolanic activity of microspheres. However, further increasing the microsphere dosage beyond 110 kg/m^3^ does not consistently improve the 28-day mechanical properties, and the resulting strength changes depend on curing temperature. Therefore, a microsphere dosage of approximately 110 kg/m^3^ may provide a reasonable balance between early-age and later-age mechanical performance under the investigated conditions. In summary, under low-temperature curing conditions, moderately reducing microsphere content is beneficial for early-age strength development, whereas under higher-temperature curing, an appropriate increase in microsphere content is favorable for stabilizing mechanical performance over the entire curing period. Nevertheless, the microsphere dosage should not exceed 110 kg/m^3^.

### 3.4. Effect of Steel Fibers on the Early-Age Mechanical Properties of Non-Steam-Curing UHPC

**Figure 4:**
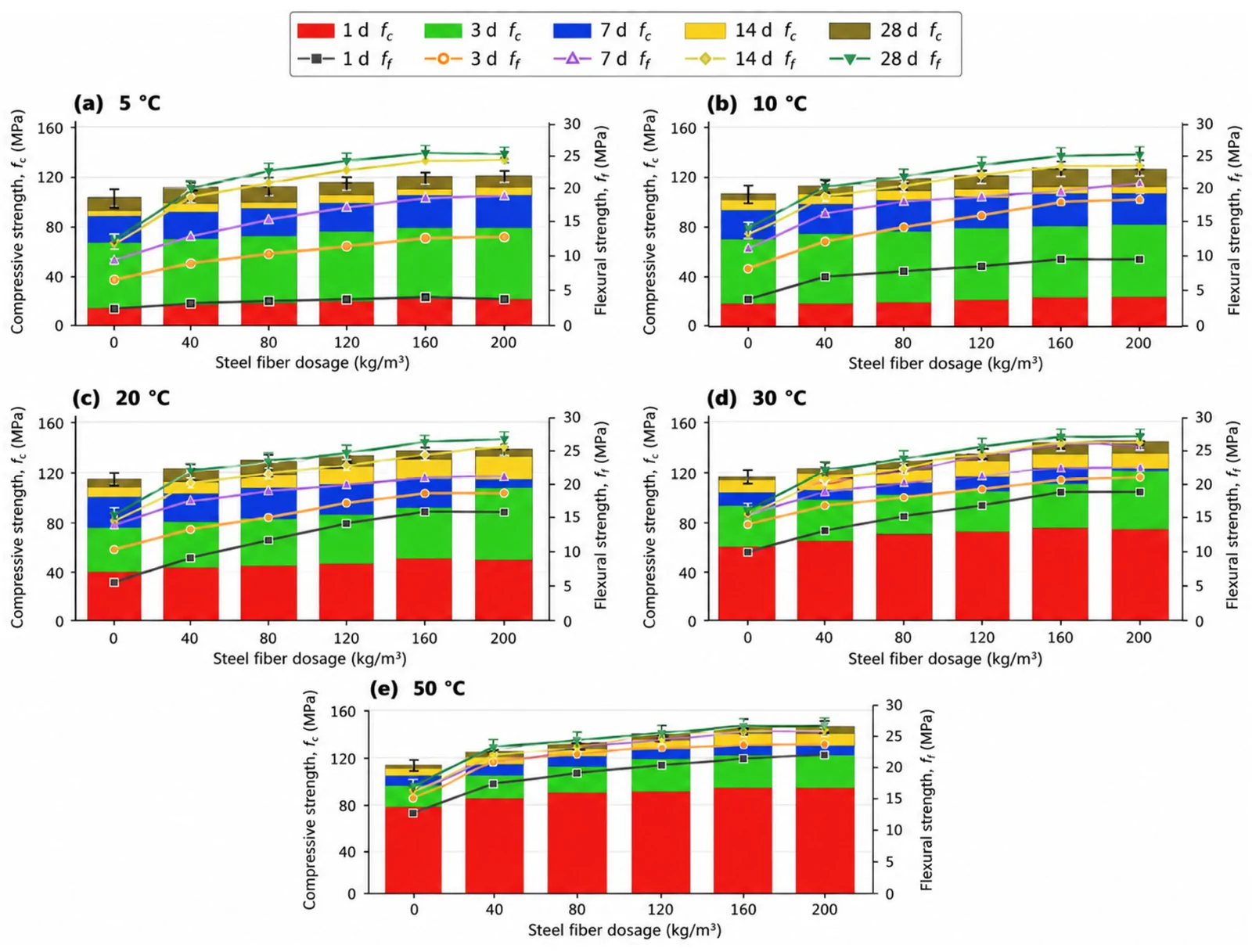
Effect of steel-fiber dosage on the mechanical properties of non-steam-curing UHPC: (**a**) 5 °C; (**b**) 10 °C; (**c**) 20 °C; (**d**) 30 °C; (**e**) 50 °C.

Figure 4 presents the effect of steel-fiber dosage on the mechanical properties of UHPC at different curing temperatures. According to Table 1 and Figure 4, increasing the steel-fiber dosage from 40 to 160 kg/m^3^ generally increased both compressive and flexural strengths across the investigated curing temperatures. Notably, the enhancement in flexural strength is generally more pronounced than that in compressive strength. This trend may be associated with the crack-bridging action of steel fibers, which can restrain crack propagation in the UHPC matrix, particularly under flexural loading. However, the orientation and spatial distribution of steel fibers were not directly quantified in this study, and their specific contributions to the observed strength changes therefore remain uncertain. At lower curing temperatures, steel fibers contribute more significantly to mid- and late-age mechanical properties, whereas at higher temperatures, their effect is more pronounced during early ages. This temperature-dependent behavior may be related to the development of the cementitious matrix during curing. At lower curing temperatures, slower early-age hydration may limit the contribution of steel fibers to mechanical strength, whereas continued matrix development with curing age may improve the effectiveness of fiber reinforcement. At higher curing temperatures, accelerated early-age hydration may contribute to more rapid matrix strength development, allowing the reinforcing effect of steel fibers to become effective at earlier ages. This interpretation is consistent with the observed strength trends, although changes in fiber–matrix interfacial bonding were not directly evaluated in this study. Additionally, regardless of curing temperature, when steel-fiber content exceeds 160 kg/m^3^, further increases provide little additional improvement in UHPC mechanical performance. Therefore, excessive fiber addition is not optimal; the fiber content should be controlled within an optimal range for balanced performance.

## 4. Early-Age Mechanical Property Prediction Models for Non-Steam-Curing UHPC

### 4.1. Time-Dependent Strength Evolution Model for the Optimized UHPC Mixture Under Different Curing Temperatures

**Figure 5:**
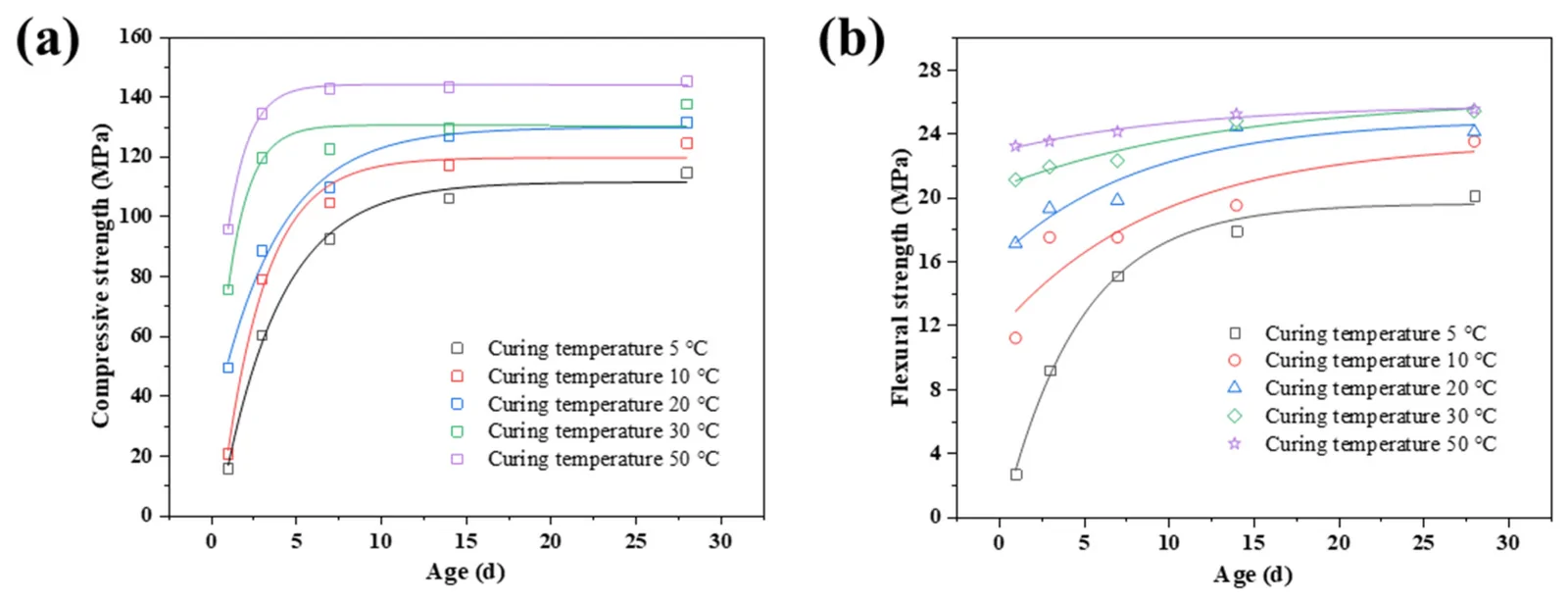
Evolution of mechanical properties of non-steam-curing UHPC with curing age under different curing temperatures: (**a**) compressive strength; (**b**) flexural strength.

Figure 5 illustrates the development of compressive and flexural strengths of the optimized UHPC mixture with curing age at different temperatures. As shown, both strengths increased rapidly at early ages, followed by a gradual reduction in the rate of strength gain as curing continued. To quantitatively describe the combined effects of curing temperature and age on UHPC strength development, an empirical temperature–time index was introduced, as expressed in Equation (1).

(1) M=∑(T+15)⋅Δt

where *M* is the empirical temperature–time index (°C·d), *T* is the curing temperature (°C), and Δ*t* is the curing time interval (d). The constant 15 °C is a numerical offset in the empirical formulation and is not intended to represent an experimentally determined datum temperature. Accordingly, the proposed index is used solely for strength prediction within the investigated temperature and age ranges.

**Figure 6:**
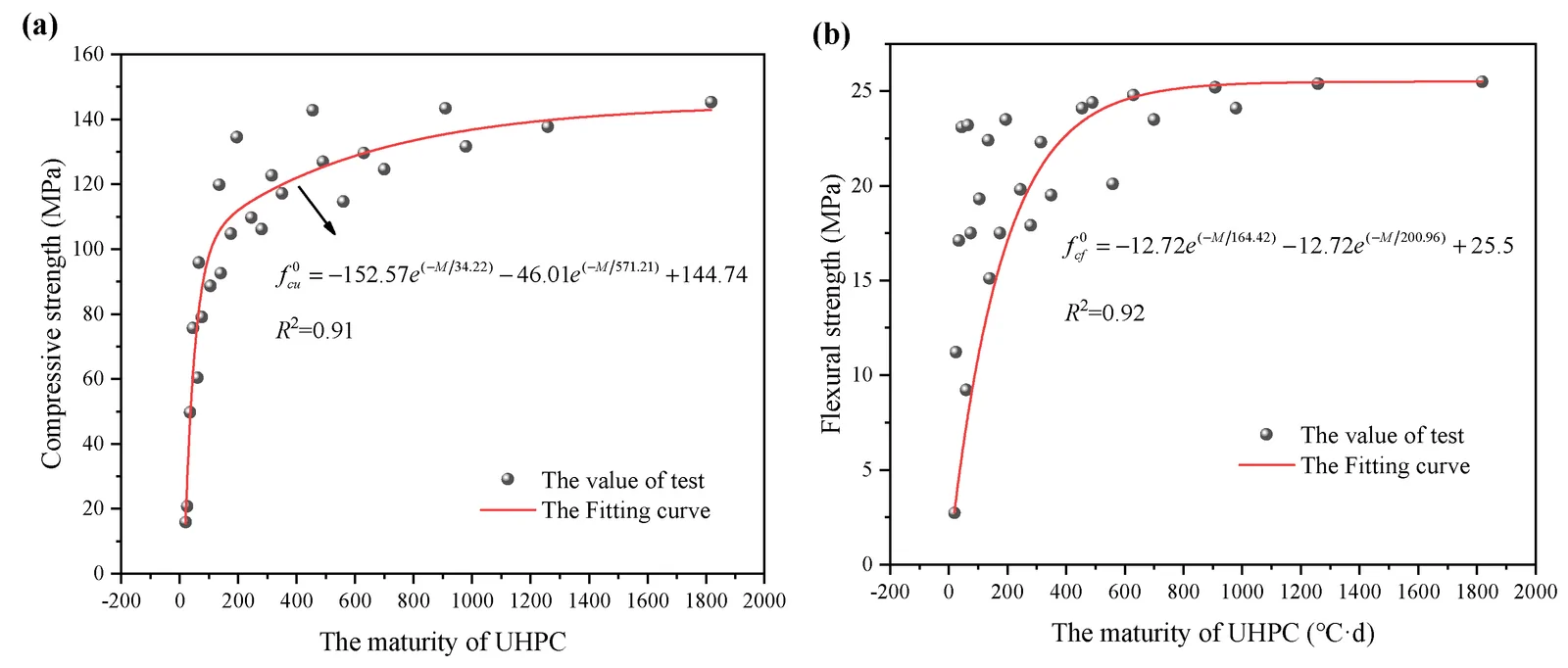
Relationship between UHPC maturity and mechanical properties: (**a**) compressive strength; (**b**) flexural strength.

Based on Equation (1), the UHPC maturity under different curing temperatures and ages was calculated and listed in Table 2. Figure 6 illustrates the relationship between UHPC mechanical properties and maturity. As shown, the relationship between compressive or flexural strength and maturity can be described by an exponential function. By fitting the data in Figure 6, the following model is obtained:

(2) $$f_{\mathrm{cu}}^{0}=-152.57e^{\left(-M/34.22\right)}-46.01e^{\left(-M/571.21\right)}+144.74$$

(3) $$f_{\mathrm{cf}}^{0}=-12.72e^{\left(-M/164.42\right)}-12.72e^{\left(-M/200.96\right)}+25.5$$

where $f_{\mathrm{cu}}^{0}$ and $f_{\mathrm{cf}}^{0}$ represent the compressive and flexural strengths of UHPC at the optimal mix, respectively, in MPa; M is the UHPC maturity, which can be calculated using Equation (1). The strength evolution models were established by fitting the experimental data obtained from five curing temperatures and five curing ages, comprising 25 temperature–age combinations. The coefficients of determination reported in Figure 6 represent the goodness of fit to the calibration data rather than independent validation performance.

### 4.2. Prediction of 28-Day Compressive and Flexural Strengths of Non-Steam-Curing UHPC Under Different Curing Temperatures and Constituent Dosages

To describe the nonlinear relationships between the 28-day mechanical strengths of UHPC and the investigated variables, second-order polynomial models were adopted. The models include linear and quadratic terms for curing temperature and constituent dosages, together with temperature–constituent interaction terms. The experimental program comprised 80 temperature–dosage conditions across the silica fume, microsphere, and steel fiber series. Based on the 28-day experimental strength data, the compressive and flexural strength models are expressed by Equations (4) and (5), respectively.

(4) $$\begin{array}{l} f_{\mathrm{cu}}=73.57+0.27G+0.14M_{b}+0.14S+0.81T-0.0013G^{2}-0.0007M_{b}^{2}\\ -0.0004S^{2}-0.018T^{2}+0.002\mathrm{GT}+0.0008M_{b}T+0.0028\mathrm{ST} \end{array}$$

(5) $$\begin{array}{l} f_{\mathrm{cf}}=10.54+0.025G+0.022M_{b}+0.082S+0.16T-0.00012G^{2}-0.000075M_{b}^{2}\\ -0.00017S^{2}-0.0012T^{2}-0.000054\mathrm{GT}+0.000046M_{b}T-0.00023\mathrm{ST} \end{array}$$

where *f_cu_* and *f_cf_* represent the 28-day compressive and flexural strengths of UHPC (MPa), respectively; *G*, *M_b_*, and *S* denote the dosages of silica fume, microspheres, and steel fibers (kg/m^3^), respectively; and *T* represents the curing temperature (°C). The coefficients of determination (R^2^) for the compressive and flexural strength prediction models were 0.91 and 0.92, respectively, indicating a good fit between the calculated and experimental values. These models are empirical and are intended for strength estimation within the investigated ranges of curing temperature and constituent dosage.

This study has several limitations. The proposed empirical models were developed using one UHPC material system under controlled laboratory curing conditions. Their predictive performance has not been independently validated using external datasets. Therefore, their applicability to other mixture systems and field curing conditions remains uncertain. Future research should consider additional UHPC mixtures, time-varying field curing histories, and independent experimental datasets to further evaluate and validate the proposed models.

## 5. Conclusions

The present study investigated the mechanical performance of UHPC under different curing temperatures and constituent dosages, revealing the influence of curing temperature, silica fume, microspheres, and steel fiber contents on the strength development and mechanical behavior of UHPC. Based on the experimental results, predictive models were established to describe the strength evolution of UHPC under varying curing conditions and mixture compositions. The main conclusions are summarized as follows:

(1) The influence of silica fume on UHPC strength depends on curing temperature and age, with a more pronounced contribution to early-age strength at elevated temperatures. Under low-temperature curing, the silica-fume dosage should be carefully selected to balance early-age and later-age mechanical performance.
(2) The effects of microsphere dosage on UHPC strength are temperature- and age-dependent. At lower curing temperatures, increasing the microsphere dosage generally reduces early-age strength. This reduction becomes less pronounced with increasing curing age and temperature. However, excessive microsphere addition does not consistently improve later-age strength, indicating the need for an appropriate dosage.
(3) Increasing the steel-fiber dosage from 40 to 160 kg/m^3^ generally improved the compressive and flexural strengths of UHPC, with a more pronounced improvement in flexural strength. This behavior may be associated with the accelerated development of the cementitious matrix at higher curing temperatures, which allows the reinforcing effect of steel fibers to become effective at earlier ages.

The proposed empirical models are applicable to the UHPC material system investigated in this study. The strength evolution models were developed for curing ages of 1–28 days and temperatures of 5–50 °C. The 28-day strength models were established using silica fume dosages of 40–180 kg/m^3^, microsphere dosages of 30–220 kg/m^3^, and steel fiber dosages of 40–220 kg/m^3^. Predictions for untested mixture combinations or conditions outside these ranges should be treated with caution.

## Figures and Tables

**Table 1:** Optimal Mix Proportion of Non-Steam-Curing UHPC [40].

Component	Dosage (kg/m^3^)	Component	Dosage (kg/m^3^)
Cement	850.56	Micron-sized MgO (4.0 μm)	22.32
Silica fume	115.20	Submicron MgO (0.4 μm)	22.32
Microspheres	105.60	SAP	2.23
Steel fibers	160.00	Effective mixing water	200.88
Medium sand	303.00	Additional internal-curing water	55.80
Fine sand	709.00	Superplasticizer	20.09

**Table 2:** UHPC maturity under different curing temperatures and ages.

	Temperatures (°C)	5	10	20	30	50
Ages (d)						
1		20	25	35	45	65
3		60	75	105	135	195
7		140	175	245	315	455
14		280	350	490	630	910
28		560	700	980	1260	1820

## Data Availability

The original contributions presented in this study are included in the article. Further inquiries can be directed to the corresponding author.
